# Eosinophil-Associated Genes are Potential Biomarkers for Hepatocellular Carcinoma Prognosis

**DOI:** 10.7150/jca.95138

**Published:** 2024-09-03

**Authors:** Qinghao Wang, Zixin Zhang, Hao Zhou, Yanling Qin, Jun He, Limin Li, Xiaofeng Ding

**Affiliations:** 1 The National & Local Joint Engineering Laboratory of Animal Peptide Drug Development, College of Life Science, Hunan Normal University, Changsha, 410081, China.; 2 Institute of Interdisciplinary Studies, Hunan Normal University, Changsha, 410081, China.; 3 Hunan Provincial Key Laboratory of Regional Hereditary Birth Defects Prevention and Control, Changsha Hospital for Maternal & Child Health Care Affiliated to Hunan Normal University, Changsha, 410007, China.; 4 College of Engineering and Design, Hunan Normal University, Changsha, 410081, China.; 5 Peptide and Small Molecule Drug R&D Platform, Furong Laboratory, Hunan Normal University, Changsha, 410081, China.

**Keywords:** hepatocellular carcinoma, eosinophils, RAMP3, G6PD, SSRP1, PLOD2

## Abstract

**Background:** Eosinophils, a type of white blood cell originating from the bone marrow, are widely believed to play a crucial role in inflammatory processes, including allergic reactions and parasitic infections. However, the relationship between eosinophils and liver cancer is not well understood.

**Methods:** Tumor immune infiltration scores were calculated using single-sample Gene Set Enrichment Analysis (ssGSEA). Key modules and hub genes associated with eosinophils were screened using Weighted Gene Co-expression Network Analysis (WGCNA). Univariate and multivariate Cox analyses, along with LASSO regression, were used to identify prognostic genes and create a risk model. The Tumor Immune Dysfunction and Exclusion (TIDE) score was used to evaluate the immunotherapeutic significance of the eosinophil-associated gene risk score (ERS) model. Experiments such as flow cytometry, immunohistochemical analysis, real-time quantitative PCR (RT-qPCR), and Western blotting were used to determine gene expression levels and the status of eosinophil infiltration in tumors.

**Results:** A risk trait model including 4 eosinophil-associated genes (RAMP3, G6PD, SSRP1, PLOD2) was developed by univariate Cox analysis and Lasso screening. Pathologic grading (p < 0.001) and model risk scores (p < 0.001) were found to be independent predictors of hepatocellular carcinoma (HCC) patient survival. Western blotting revealed higher levels of eosinophil peroxidase (EPX) in HCC tissues compared to adjacent normal tissues. Immunohistochemistry showed that eosinophils mainly infiltrated the connective tissue around HCC. The HCC samples showed low expression of RAMP3 and high expression of G6PD, SSRP1, and PLOD2, as detected by IHC and RT-qPCR analysis. The *in vivo* mouse experiments showed that IL-33 treatment induced the recruitment of eosinophils and reduced the number of intrahepatic tumor nodules.

**Conclusion:** Overall, eosinophil infiltration in HCC is significantly correlated with patient survival. The risk assessment model based on eosinophil-related genes serves as a reliable clinical prognostic indicator and provides insights for precise treatment of HCC.

## Introduction

HCC accounts for 80-90% of primary liver cancers, and despite advances in screening and treatment technologies, the mortality rate for HCC continues to rise 1. The prognosis for HCC patients remains unsatisfactory, and the 5-year survival rate was less than 18% 2. Although surgery is considered the most effective means of treating cancer, only 5-15% of liver cancer patients meet the criteria for surgical resection 3. Recently immunotherapy has emerged as a promising way for HCC therapy. These include clinically administered targeted PD-L1/PD-1 inhibitors and anti-CTLA4 antibodies 4. However, the problem with immunotherapy is that only a small proportion of HCC patients achieve a sustained response 5. Therefore, there is a clinical need for new approaches to identify patients who respond to immunotherapy in order to further increase the benefit of immunotherapy for patients.

Pathologic staging and the Barcelona Clinic Liver Cancer (BCLC) staging system are commonly used to predict patients' prognosis 6, 7, but clinical studies have shown that patients may have different risk of recurrence and death even if they belong to the same pathologic grade and stage 8. The development and progression of malignant tumors are complicated processes in which various internal and external factors crosslink. Therefore, we can construct prognostic models from multiple perspectives to select and evaluate treatment methods targeting immunological, metabolic, epigenetic, and tumor microenvironment-related biomarkers 9-11.

Eosinophils are granulocytes induced by GATA-1 from bone marrow hematopoietic stem cells 12, participating in tissue homeostasis and repair, parasite clearance, as well as the pathophysiology of various diseases, including allergic asthma and autoimmune diseases 13. Many tumors are accompanied by infiltration of eosinophils in the tissues and increased levels in the blood. However, the role of eosinophils appears to differ across various tumors, they can either have antitumor effects or promote tumor progression 14, 15. During the occurrence and development of malignant tumors, the production of eotaxin-1/CCL11 16, 17, IL-5, eotaxin-2/CCL24 and RANTES/CCL5 can activate the chemokine receptors CCR3 and CCR1 on eosinophils 18, 19. This activation promotes the migration and recruitment of eosinophils into the tumor microenvironment (TME) and activates eosinophils. In a study on colorectal cancer, eosinophils were found to indirectly promote the recruitment of CD8^+^ T cells into the TME by secreting chemokines, and they stimulate macrophages towards a pro-inflammatory (M1) phenotype 20. The direct contact between human eosinophils and natural killer (NK) cells leads to an upregulation of NK cell effector functions and thus to an increased production of interferon (IFN)-γ. Recent studies have shown that increased eosinophil levels during ICB therapy are associated with responses to PD-1, PD-L1, or CTLA-4-targeted antibodies in patients with metastatic melanoma 21, 22, renal cell carcinoma 23, and non-small cell lung cancer 24. However, the relationship between the development of HCC and eosinophil infiltration has not yet been clarified.

Here, we found that eosinophil infiltration is associated with the prognosis of HCC patients. To explore additional prognostic biomarkers related to immune cells, we applied WGCNA to identify genes associated with eosinophil infiltration and used LASSO, univariate and multivariate Cox regression, to select four genes (RAMP3, G6PD, SSRP1, and PLOD2). We then established a prognosis model based on eosinophil infiltration. The model was validated against the GSE14520 database and experimental expression analysis and showed that it is able to stratify the risk of cancer patients. The model is also capable of distinguishing patient populations that respond to immunotherapy. The *in vivo* experiments have shown that IL-33-mediated eosinophil recruitment therapy can significantly control the number of liver tumor nodules in mice. Furthermore, we found that IL-33-mediated recruitment of eosinophils is also associated with the infiltration of CD8^+^ T cells and macrophages. The study reveals the effect of eosinophil infiltration on the development of HCC, constructs a risk model based on eosinophil-associated genes and suggests that this is important to improve the efficacy of personalized treatment for HCC patients.

## Methods and materials

### Data downloading and filtering

The mRNA expression data and clinical information used for the analysis were downloaded from the TCGA-LIHC database and the GEO database GSE14520. The former contains 379 HCC samples and 59 adjacent normal samples, while the latter contains 241 tumor samples and 52 adjacent non-tumor samples. Prior to data analysis, samples from patients who had undergone chemotherapy were excluded to avoid potential effects of chemotherapy on the patients' immunologic microenvironment. The analyzed data excludes samples with missing survival information and samples with a survival time of less than 30 days. Finally, the analysis is performed on 327 tumor samples from the TCGA-LIHC database. Data for all boxplots and correlation plots were derived from RNAseq data in fragments per kilobase of transcript per million mapped reads (FPKM) format from the Liver Hepatocellular Carcinoma (LIHC) project. Subsequently, the RNAseq data in FPKM format were transformed into log2 format. R software (version 3.6.3) was used to generate the box plots and correlation plots. TIDE analysis of the TCGA-processed data was downloaded to predict the potential response to immune checkpoint blockade in HCC 25.

### The landscape of immune cell infiltration

Single-sample Gene Set Enrichment Analysis (ssGSEA) allows the calculation of enrichment scores for each sample and gene set 26. In this analysis, 28 immune gene sets from the TISIDB were used as marker genes for immune cells 27. The ssGSEA algorithm, implemented using R packages, was then used to assess immune infiltration scores for each sample. Scores were calculated for the 28 immune-related cell types in each sample. Survival algorithms in R were then used to assess the relationship between the score of each immune cell type and patient survival, with a significance level of p < 0.05.

### Weighted gene co-expression network analysis

The hierarchical clustering algorithm (hclust) was used to remove outlier samples, finally leaving the gene expression matrix with 300 samples. The R package WGCNA was applied to calculate the gene expression matrix for the selected 5000 representative genes from the TCGA-LIHC center. The optimal range for setting the soft threshold power (β-value) is between 1 and 20, and the pickSoftThreshold function was used to calculate the optimal power value and construct the adjacency matrix. By considering connectivity, it can be ensured that the distribution of genes corresponds to a scale-free network 28.

After obtaining the Topological Overlap Matrix (TOM) from the gene expression data, hierarchical clustering is used again to cluster the genes. The minimum size of gene modules is set to 50, and the threshold for merging modules is set to 0.15. Subsequently, the clustered gene results are divided to acquire distinct gene modules. To perform the correlation analysis between module characteristic genes and sample traits, a Pearson correlation test was performed. Then, the eosinophils are focused and the two modules that correlate most strongly with eosinophils were extracted for further analysis (p<0.05).

### Lasso-Cox regression analysis

We found two modules containing 1039 genes that were correlated with eosinophils by WGCNA analysis. We combined these modules with the survival data and performed a univariate Cox analysis with gene expression levels as independent variables (p<0.001). To select the genes, we used the LASSO-Cox regression method, which prevents overfitting of the model and fine-tunes the model to avoid excessive compression of the coefficients. Ultimately, 4 genes were selected. Using the R package “My.stepwise”, we performed a stepwise regression analysis to select the optimal gene combination for multivariable regression analysis. Their coefficients were extracted to calculate a risk score for each patient. The formula for calculating the eosinophil-associated gene risk score (ERS) model is as follows:



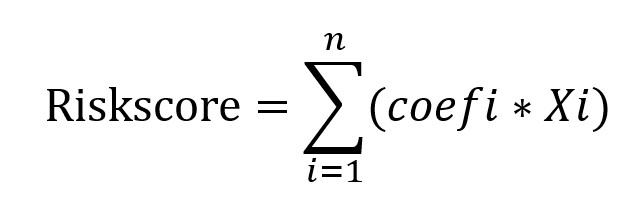



In the formula for calculating the risk, "X" stands for the mRNA level of the gene, and "Coefi" for the coefficient of the gene selected. The same calculation is also applied to the validation cohort in the GSE14520 database.

### Characteristic analysis of the eosinophil-related risk prediction model

According to the risk assessment formula, each sample is assigned a risk assessment score. Patients with risk assessment scores above the median are categorized as the high-risk group, while patients with scores below the median are categorized as the low-risk group. Kaplan-Meier survival curves are generated using survival analysis algorithms in R to compare the differences in prognosis between the two subgroups. In addition, ROC curves are plotted to show the performance of the risk model. Univariate and multivariate Cox regression analyses are then performed to confirm whether ERS is a significant independent factor for the prognosis of patients with HCC.

The expression of immune checkpoint-related genes and the clinical characteristics of patients in the high-risk and low-risk group are compared. This analysis evaluates the prognostic significance of the risk score, examines its independent impact on the survival of HCC patients, and explores the relationship between the expression of immune checkpoint-related genes and clinical characteristics in different risk groups.

### Validation of the risk prognosis models

Based on the risk scoring model of the training dataset, the GSE14520 dataset was divided into high-risk group and low-risk group. A ROC curve was plotted to assess performance. Univariate Cox regression analysis was then performed to show that ERS is a meaningful independent prognostic factor for HCC patients 29. An integrated column chart was constructed, combining the risk features with other clinicopathological characteristics. Finally, the performance of the nomogram was validated by plotting calibration curves.

### Functional enrichment analysis

To calculate the logFC (log-fold change) value, we performed enrichment analysis using the clusterProfiler R package for Kyoto Encyclopedia of Genes and Genomes (KEGG) pathways and Gene Ontology (GO), with a P threshold of < 0.05. Using the KEGG database, we analyzed the significantly enriched pathways in low and high risk populations using Gene Set Enrichment Analysis (GSEA) software 30, with a threshold P-value < 0.05.

### Cell culture and intrahepatic mouse model

The authenticated murine hepatocellular carcinoma cell line Hepa1-6 was purchased from Servicebio. Cells were cultured in Dulbecco's Modified Eagle Medium (DMEM, Thermo Science, USA) supplemented with fetal bovine serum (FBS, Thermo Scientific), penicillin G, and streptomycin (Invitrogen Life Technologies, USA). Cells were maintained in a humidified atmosphere containing 5% CO_2_ at 37˚C. 1×10^6 tumor cells were injected into the left hepatic lobe of randomly selected male C57BL/6 mice aged 6-7 weeks. Three days after injection, recombinant IL-33 (rIL-33, PeproTech, USA) or PBS were administered intraperitoneally (IP) at a dose of 0.4 μg per mouse, three times a week for two consecutive weeks. After 17 days, mice were sacrificed and tumor weight and volume were recorded, followed by tumor tissue analysis.

### Flow cytometry analyses

To detect eosinophils recruitment, mice were intravenously injected with rIL-33 or PBS at a dose of 0.5 μg per mouse at 10:00 am and 7:00 pm. To isolate liver and lung parenchymal cells from mice, liver was perfused with HBSS buffer containing EDTA at 37°C for 5 min the following day at 14:00, followed by perfusion with 0.5 mg/mL collagenase IV (Coolaber, China) for 10 min. Cells were then isolated with 35% Percoll at 1.04 g/mL (Sigma-Aldrich, USA). The liver tissue was then incubated with NH_4_ lysis buffer at room temperature for 3 min to remove red blood cells, and the single cell suspensions were obtained by passed through a 70 μm filter. Cells were resuspended in PBS solution containing 1% BSA and stained with antibodies targeting various markers on ice and in the dark for 40 min. Anti-CCR3-APC (144511) and anti-Siglec F-PE (155505) antibodies were purchased from BioLegend, and anti-CD45-FITC (30-F11) from Elabscience. Flow cytometry analysis was performed using a MoFlo Astrios EQ flow cytometer (Beckman-Coulter, USA). Data analysis was carried out using FlowJo v10.8 software.

### Real-time quantitative PCR analysis

Total RNA was extracted from HCC tissues using TRIzol reagent (Thermo Fisher Scientific), and reverse transcription to cDNA was performed using MMLV reverse transcriptase and random primers (Sangon, China). RT-qPCR based on SYBR Green (Invitrogen) was performed using the ABI 7900 thermal cycler (Thermo Fisher Scientific) 31. The reaction mixture was incubated at 95°C for 5 min, followed by 15 sec at 95°C and 20 seconds at 60°C. The PCR primers are listed in Supplementary Table 1. The relative expression values of the genes compared to the controls were calculated using 2^-ΔΔCt^.

### Western blots

HCC tissues were homogenized and lysed in RIPA buffer 32. The primary antibodies are rabbit polyclonal antibodies against EPX (bs-3381R, 1:2000, Bioss) and mouse monoclonal antibodies against GAPDH (AP0063, 1:5000, Abbkine). The secondary antibodies were HRP-conjugated goat anti-mouse/rabbit antibodies from ABclonal. The grayscale values were analyzed using Image J software.

### Immunohistochemical (IHC) analysis and immunofluorescence staining

The experiments obtained approval from the Ethics Committee of Hunan Normal University and informed consent from involved patients. For IHC analysis, the HCC tissues were fixed with formaldehyde and embedded in paraffin (FFPE) as described 31, 33. The primary antibodies are rabbit polyclonal antibodies against EPX, RAMP3 (bs-11972R, 1:2000, Bioss), G6PD (R26802, 1:1000, Zenbio), SSRP1 (ER1901-13, 1:1000, Huabio) and PLOD2 (bs-12731, 1:1000, Bioss).

For immunofluorescence staining, the primary antibodies used are anti-CD4 (GB11064, 1:1500), anti-CD8 (GB15068, 1:2000), and anti-F4/80 (GB113373, 1:4000) from Servicebio. Each primary antibody was incubated overnight at 4°C. After incubation, the slides were washed and incubated at room temperature for 50 min with HRP-conjugated secondary antibodies. Tyramide signal amplification reagents (TSA, G1223/G1231, Servicebio) were then applied to amplify the signals for 10 min, which was repeated after incubation with the second primary and secondary antibodies. Finally, the slides were counterstained with DAPI to visualize the nuclei and the sections were mounted, followed by observing using an upright fluorescence microscope (Nikon ECLIPSE C1, Japan).

### Statistical analysis

Calculations and statistical analyses were performed using R software version 4.2.2. The Wilcoxon rank-sum test and the Kruskal-Wallis test were used for comparisons between two groups and more than two groups, respectively. The Kaplan-Meier method and the log-rank test were used for survival analysis. LASSO, univariate, and multivariate Cox regression were performed to create the prognostic model for HCC. The correlation of key genes was compared using the Pearson method. A p-value of 0.05 or less is statistically significant.

## Results

### Eosinophil infiltration is associated with the prognosis of HCC

**Figure 1:**
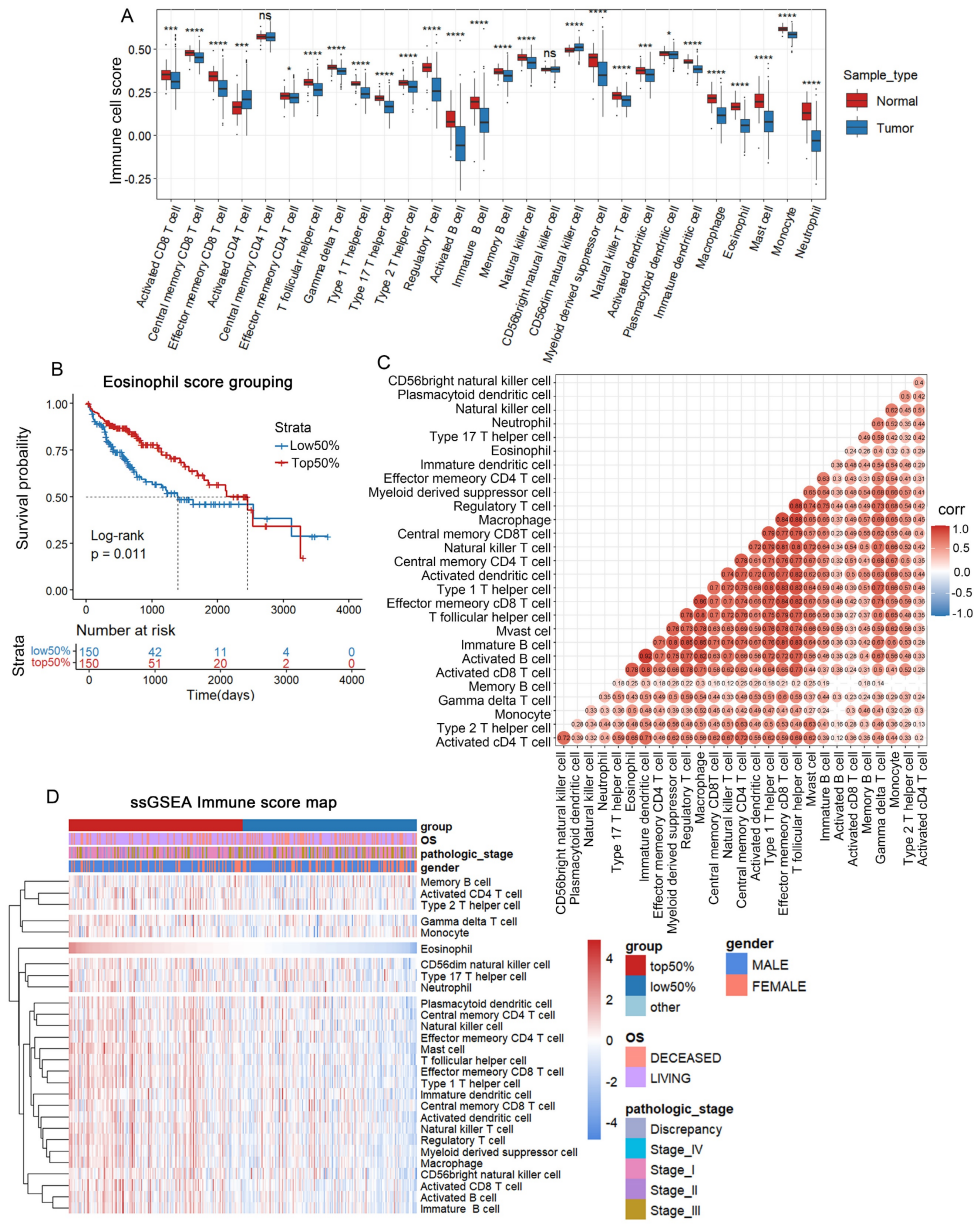
** Correlation between eosinophil infiltration and patient survival.** (A) ssGSEA immune infiltration scores for 28 immune cells in TCGA liver cancer database; (B) Kaplan-Meier survival curve for eosinophil infiltration scores; (C) Correlation between 28 immune cells by TCGA-LIHC; (D) Heatmap grouped by eosinophil infiltration scores. *, P < 0.05; **, P < 0.01; and ***, P < 0.001.

To present the infiltration of various immune cells in HCC, we used the ssGSEA algorithm to calculate the status of immune infiltration for each sample in the TCGA-LIHC dataset. The calculation revealed that the immune infiltration in adjacent tissues was generally higher than in HCC tissues (Figure 1A). Among the 28 types of immune cell infiltration scores, we found that the infiltration scores of eosinophils, activated CD8 T cells, and immature dendritic cells were correlated with patient survival (P < 0.05) (Figure 1B and Supplementary Figures 1A, B). Patients with higher infiltration of eosinophil and activated CD8 T cells tended to have longer survival, while lower infiltration of immature dendritic cells was associated with better survival. Previous reports have shown limited research on eosinophil infiltration in HCC and its impact on patient prognosis 34-36. Using the ESTIMATE R package, we calculated the tumor purity of the samples and found a negative correlation between eosinophil infiltration and tumor purity scores (Supplementary Figure 1C). Eosinophils showed no correlation with memory B cells and monocytes but showed correlations with 25 other immune cell types (Figure 1C). Based on the eosinophil infiltration score, TCGA-LIHC tissues were then divided into two subgroups for further analysis (Figure 1D). These data suggest that high eosinophil infiltration is positively correlated with HCC patient survival.

### The four genes RAMP3, G6PD, SSRP1 and PLOD2 were selected to construct the risk model

**Figure 2:**
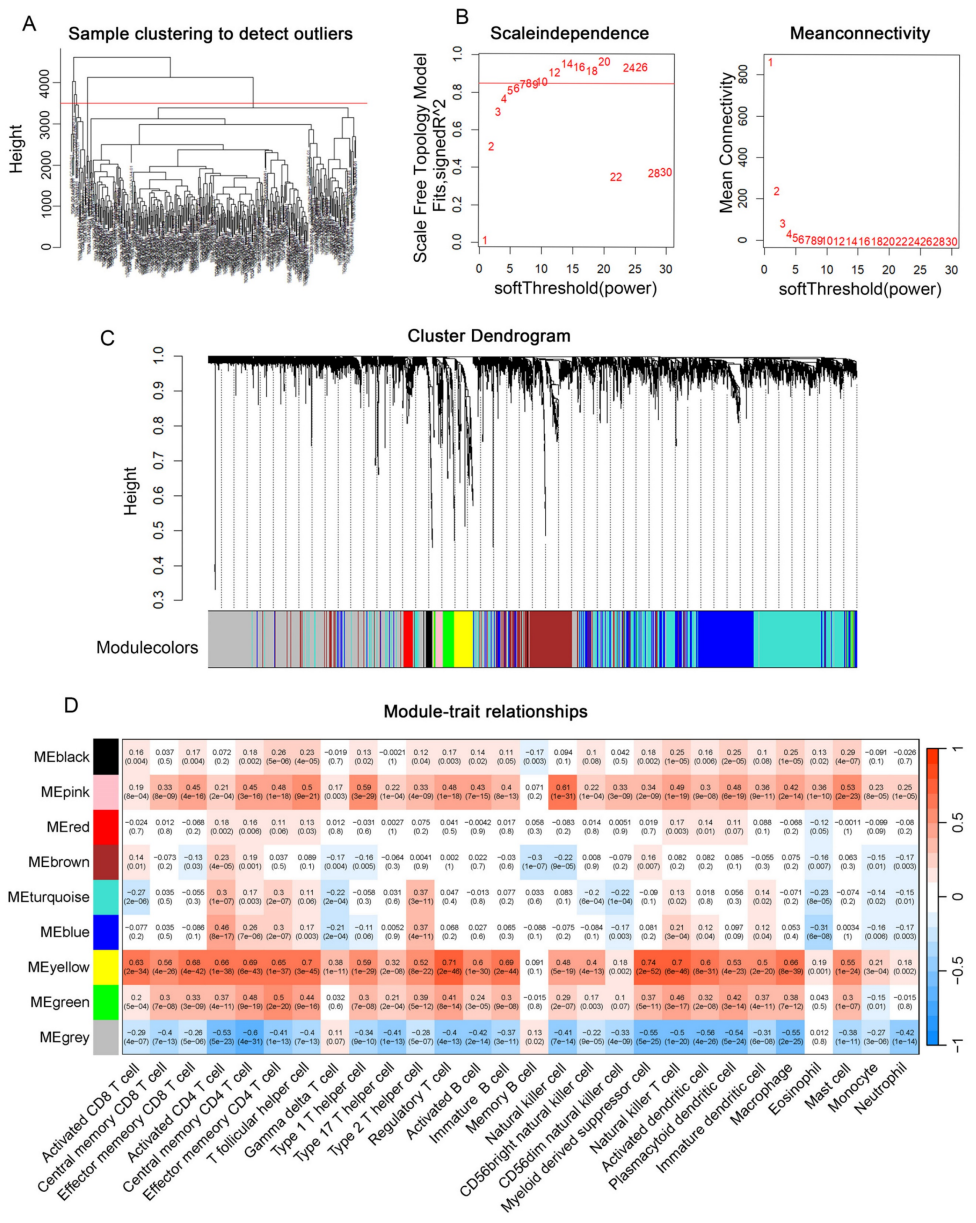
** Screening of genes associated with clinical features Using WGCNA.** (A) Application of the hclust algorithm to remove outliers. (B) Scale independence and average connectivity. (C) Gene clustering dendrogram identifying a total of 8 modules. (D) Heatmap showing the correlation between modules and clinical features, with each cell containing the corresponding correlation and P-value.

Next, we applied the hclust algorithm to remove outliers and obtained 300 liver cancer samples. The clinical characteristics of the included HCC patients are summarized in Supplementary Table 2. The pickSoftThreshold function determined the optimal power value of 8. Within each module, a minimum of 50 genes was set, and an adjacency matrix was constructed, resulting in the identification of 8 modules (Figures 2A-C). And the MEblue and MEpink modules had the highest correlation with clinical features (Figure 2D). Therefore, we selected these two modules for further analysis.

**Figure 3:**
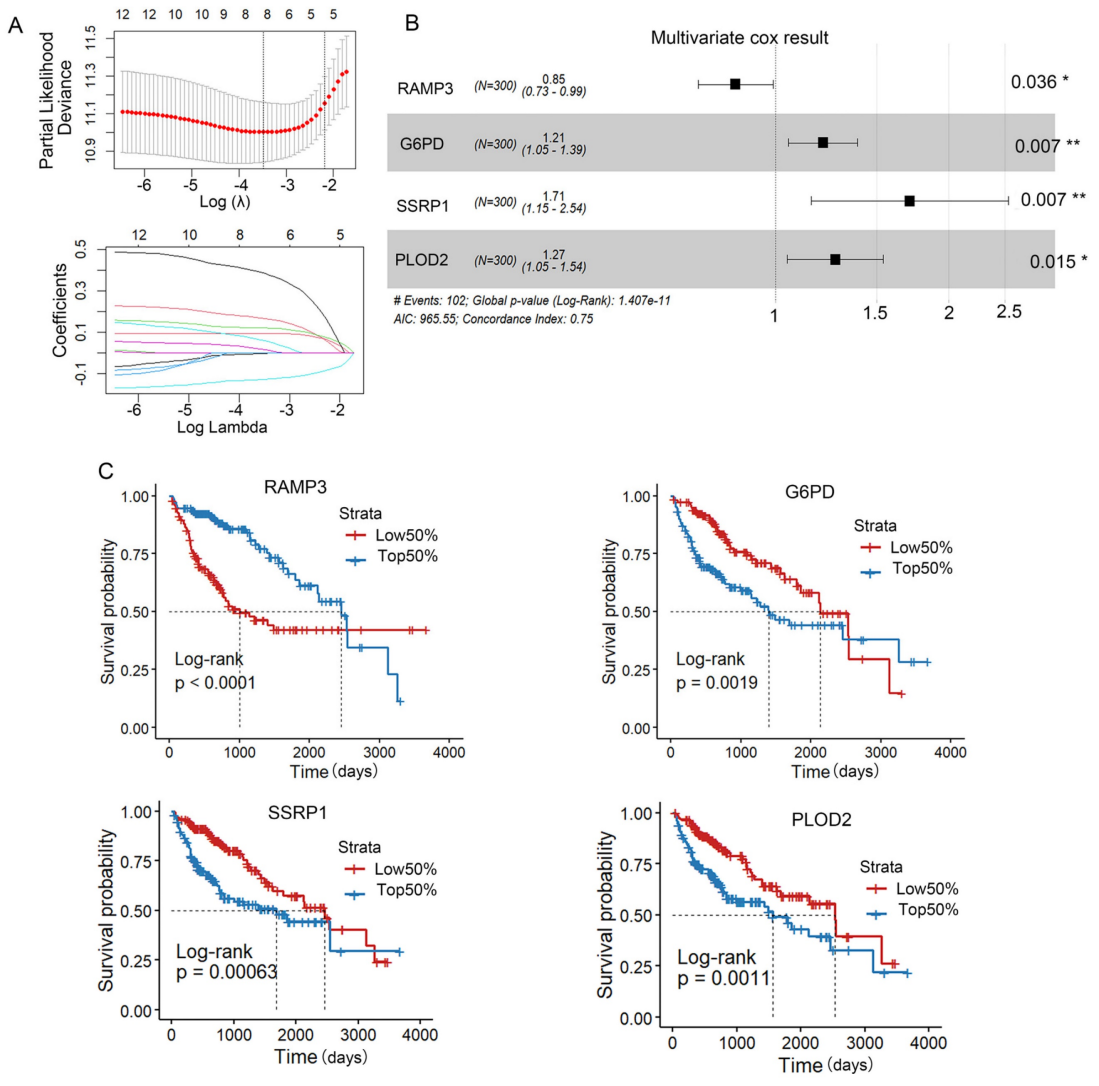
** Construction of the ERS risk assessment model.** (A) Lasso analysis was performed to find 4 genes related to patient survival. (B) Univariate COX regression analysis. (C) Kaplan-Meier survival curves for RAMP3, G6PD, SSRP1 and PLOD2 in the TCGA-LIHC database. *, P < 0.05; **, P < 0.01; and ***, P < 0.001.

Univariate Cox regression analysis showed that 215 genes associated with patient survival (p<0.001) were screened. Lasso analysis was used to narrow the selection to 11 genes (Figure 3A). We then used the My.stepwise algorithm to determine the optimal combination for risk assessment and performed multivariable Cox regression analysis to calculate the weight of each gene expression in relation to prognostic risk (Figure 3B). Finally, four genes (RAMP3, G6PD, SSRP1, PLOD2) were selected to construct a prognostic risk assessment model as eosinophil-associated genes. And RAMP3 showed a positive correlation with the favorable prognosis of patients, while the expression of G6PD, SSRP1 and PLOD2 was inversely correlated with the favorable prognosis (Figure 3C). The ERS calculation is as follows:

ERS= -0.159737× RAMP3+ 0.190134× G6PD+ 0.537636× SSRP1+ 0.238630× PLOD2

Statistical analysis revealed that the patients with an increase in ERS tended to have higher tumor grades (Supplementary Figure 1D). The HCC samples were then divided into a low-risk group and a high-risk group based on ERS.

### Construction of the Prognostic Nomogram and validation of the ERS-based risk model

**Figure 4:**
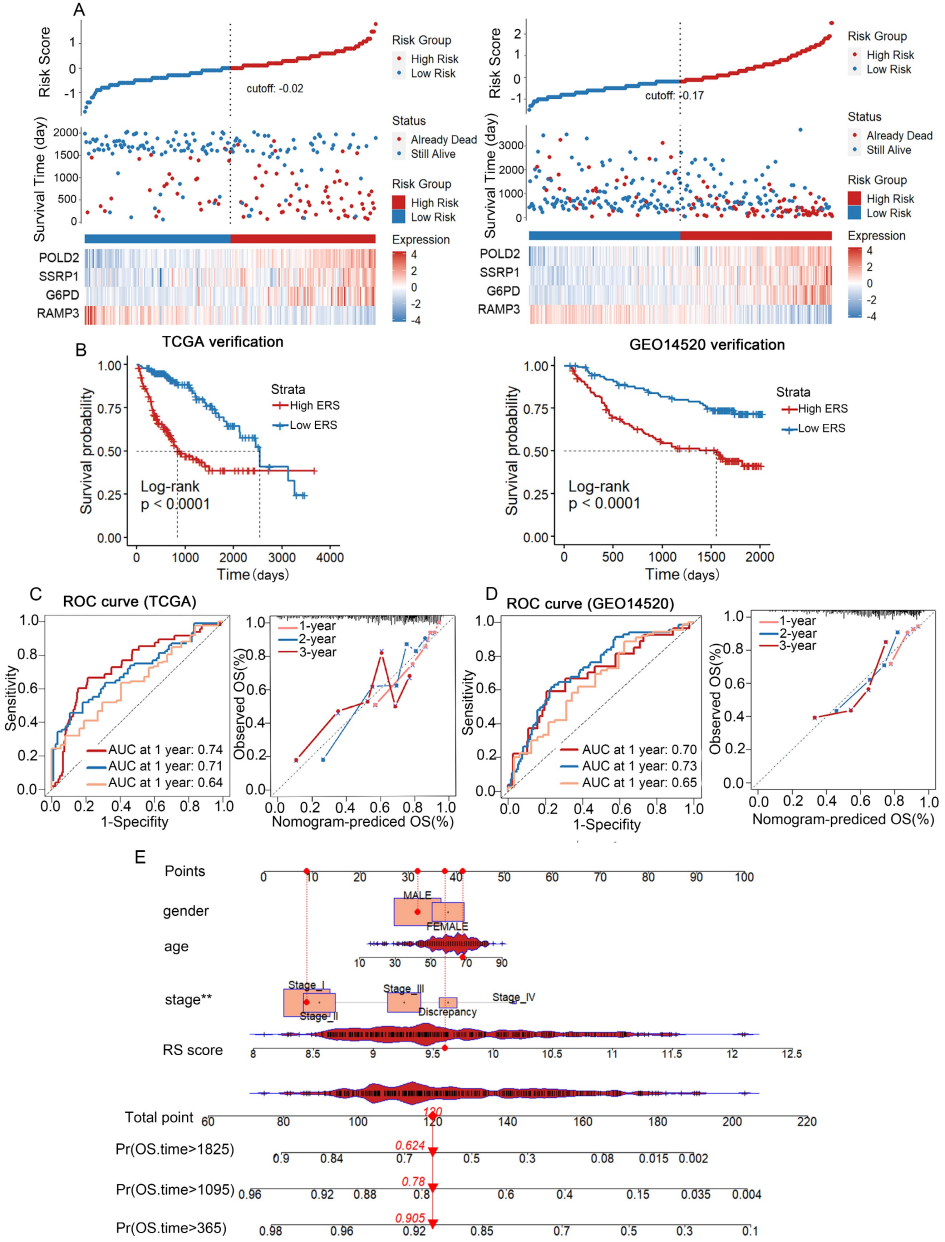
** Validation of the risk prognostic model in TCGA and GSE14520 Databases. (**A) HCC patients from TCGA and GSE14520 databases categorized into high-risk group and low-risk group based on median ERS. (B) Kaplan-Meier survival curves for high-risk group and low-risk group in TCGA and GEO databases. (C) TCGA-LIHC database: ROC curves predicting 1-year, 3-year, and 5-year survival rates with ERS, and calibration curves of the column chart. (D) ROC curves predicting 1-year, 3-year, and 5-year survival rates with ERS, and calibration curves of the column chart in GEO database. (E) Nomogram combining common clinical parameters with ERS.

**Figure 5:**
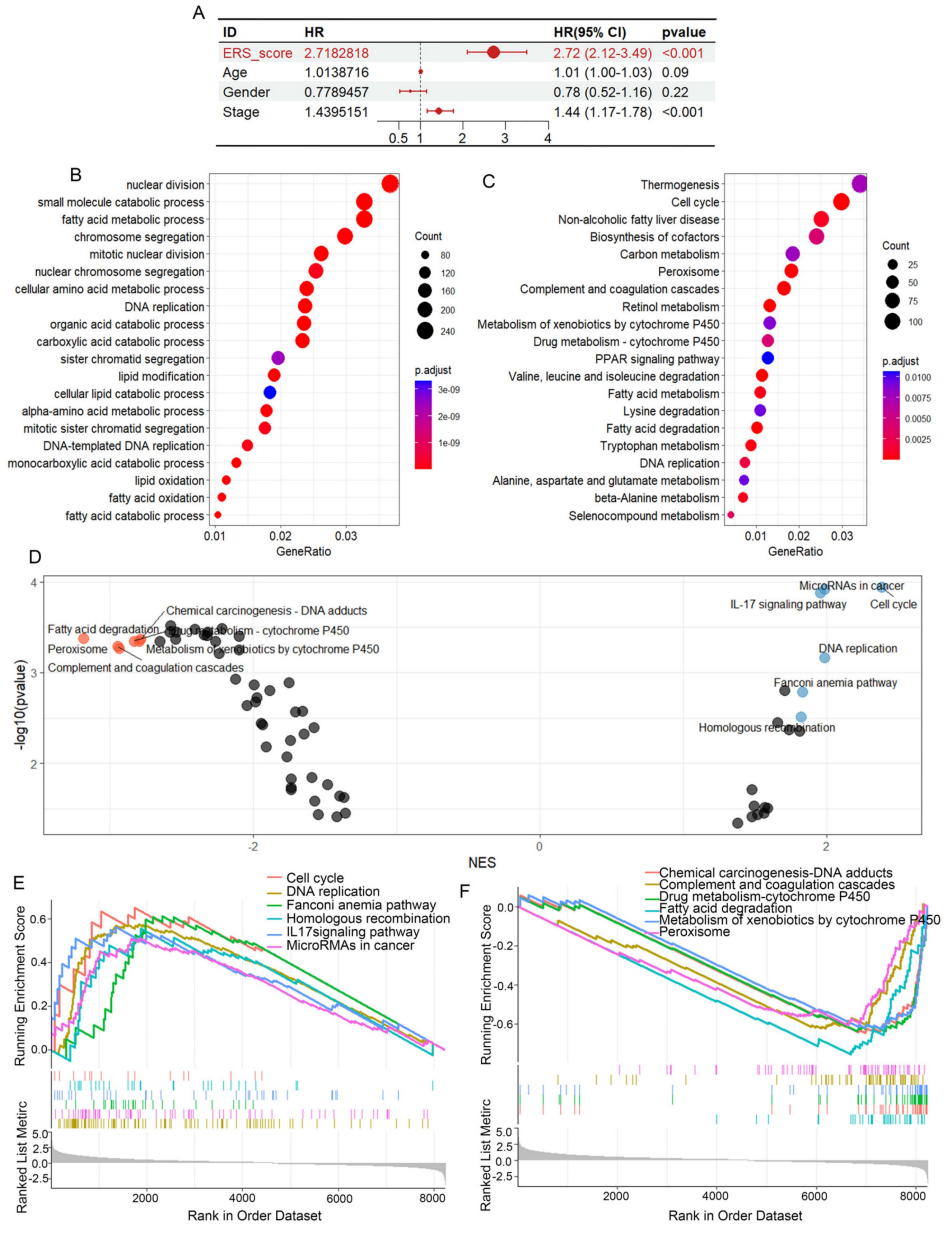
** Differential gene enrichment analysis.** (A) Univariate Cox regression analysis of ERS, age, gender, and pathological grading. (B) Based on the ERS, patients were divided into high-risk and low-risk groups. Bubble charts for GO analysis. (C) Bubble charts for KEGG analysis. (D-E) GSEA analysis showing six significantly enriched pathways in the high-risk and low-risk groups.

We used TCGA-LIHC as the training cohort while GSE14520 as the validation cohort. Based on the median risk score, HCC patients were divided into a low-risk group and a high-risk group (Figure 4A). The distribution of risk scores in the training cohort and the visualization of high-risk and low-risk patients in the validation cohort are shown in Figure 4. Kaplan-Meier analysis showed that in both the TCGA-LIHC and GSE14520 cohorts, the low ERS group had longer OS than the high ERS group (Figure 4B). The model we constructed can distinguish high-risk patients in the HCC cohort. The ROC analysis revealed that the one-year AUC value for the training group (TCGA-LIHC) was 0.819 while the one-year AUC value for the validation group (GSE14520) was 0.692 (Figures 4C, D). The eosinophil infiltration-based risk model provides a higher overall AUC in prediction compared with the single gene risk prediction models (Supplementary Figures 2A-D), multi-gene models have better prognostic performance than single-gene models, suggesting that our nomogram has good accuracy in predicting survival rates. The calibration plot of the nomogram displayed good consistency with observed probabilities (Figures 4C, D). Multivariate Cox analysis revealed that tumor stage and ERS are independent risk factors influencing the prognosis of patients with HCC (Figure 5A). Then, we constructed a prognostic nomogram consisting of gender, RS, tumor grade and clinical stage to quantitatively predict the prognosis and survival rate of HCC patients (Figure 4E). For example, a 68-year-old male was diagnosed with Stage I HCC, with a high-risk score of 9.601378. Then we can calculate the patient′s total score as 120, which corresponds to survival rates of 0.905, 0.78, and 0.624 at 1 year, 3 years, and 5 years, respectively. Taken together, these results suggest that our constructed nomogram has significant prognostic value for HCC patients and predicts the risk score of HCC patients based on various parameters.

### Gene pathway enrichment results based on ERS

To better understand the gene pathway enrichment for eosinophil infiltration and associated gene expression, we performed GO and KEGG analysis by grouping based on gene expression levels, eosinophil infiltration scores, and ERS scores. Gene enrichment analysis revealed that the four genes were related to pathways such as the cell cycle, DNA replication, nucleotide repair and amino acid excision metabolism (Supplementary Figures 3A-D), suggesting that the expression of these genes is associated with tumor progression. To investigate the differences in gene function and signaling pathways between the ERS subgroups, we then performed GO analysis and KEGG analysis. The results of GO analysis revealed that the differential genes were primarily enriched in small molecule catabolism, fatty acid metabolism and cell mitosis (Figure 5B). The results of KEGG analysis showed that the differentially expressed genes (DEGs) were enriched in pathways such as cell cycle, non-alcoholic fatty liver disease, cofactor biosynthesis, carbon metabolism, and amino acid metabolism (Figure 5C). The high ERS group exhibited higher activity in signaling pathways such as DNA replication, cell cycle, homologous recombination and microRNAs in cancer, while the low ERS group showed higher activity in signaling pathways such as chemical carcinogenesis-DNA adducts, fatty acid degradation and drug metabolism (Figures 5D-F).

GO analysis and KEGG analysis revealed that differential genes between the high/low eosinophil infiltration group were enriched in aspects related to T cell activation, T cell division and differentiation (Supplementary Figures 4A, B), suggesting a potential correlation between eosinophil infiltration and T cell activity. These results indicate that the ERS model is closely linked to major pathways involved in tumor progression.

### The ERS model can predict response to immunotherapy

**Figure 6:**
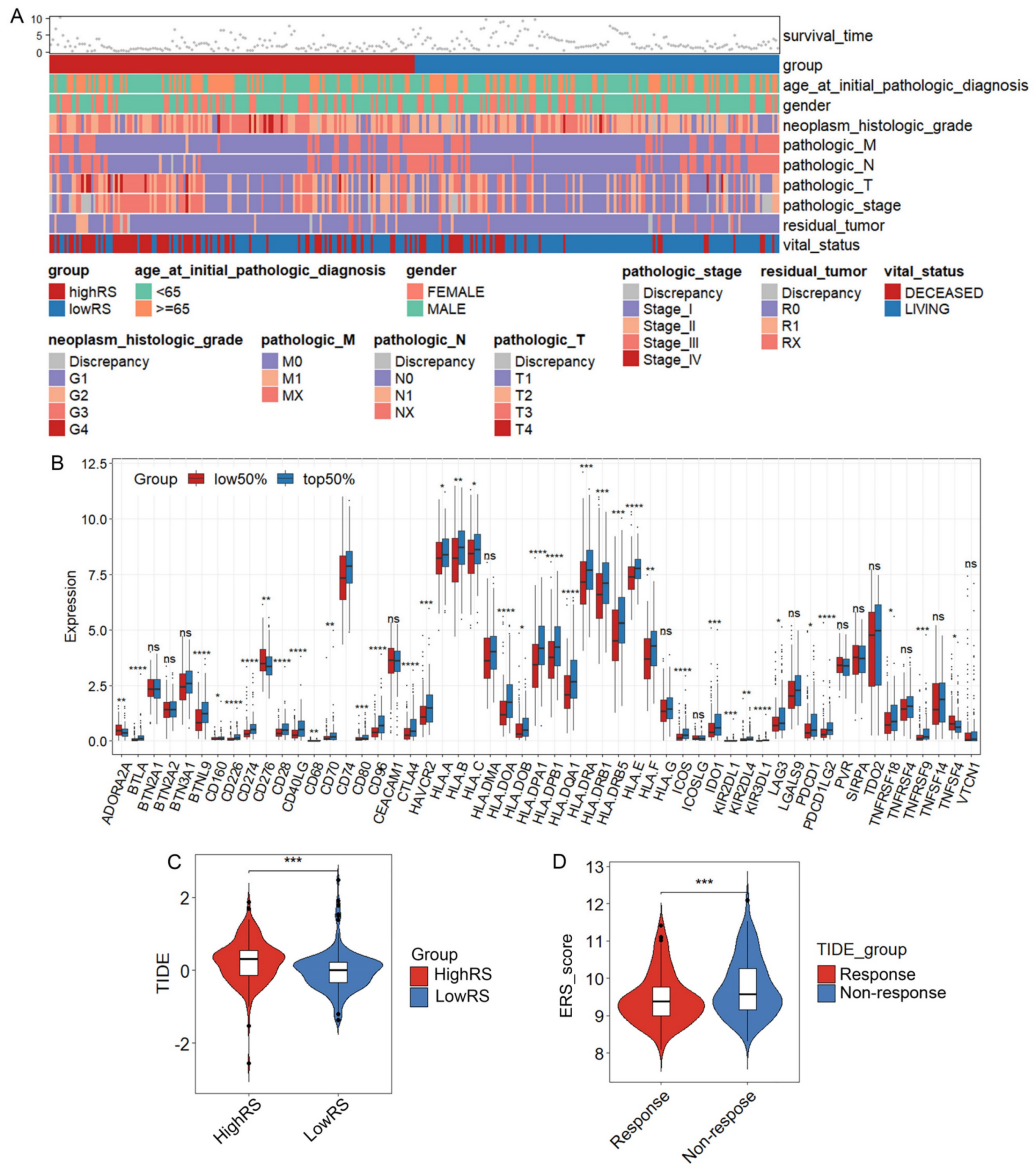
** The ERS model and immune therapy response.** (A) Heatmap of the distribution of clinical case features in the ERS high group and ERS low group patients; (B) Relationship between eosinophil infiltration and expression of genes related to immune therapy response. (C) Comparison of TIDE prediction scores between low-risk and high-risk groups; (D) Comparison of TIDE predicted immune therapy response group and non-response group ERS scores. *, P < 0.05; **, P < 0.01; and ***, P < 0.001.

To visualize the distribution of clinical variables in the low/high ERS subgroups, R package ComplexHeatmap analysis was performed in Figure 6A. The clinical subtyping scores of the low-risk and high-risk groups based on gender, tumor grade and clinical stage are shown in Supplementary Figures 4C-K. Since the eosinophil score correlated with the scores of other immune cells, TCGA-LIHC data analysis revealed a significant correlation between the eosinophil infiltration score and the expression of immune checkpoint genes, suggesting that eosinophil infiltration may contribute to the efficacy of immunotherapy (Figure 6B).

To explore whether the ERS model can distinguish patients undergoing immunotherapy, we analyzed the relationship between eosinophil infiltration and the expression of some genes related to immune checkpoints. Consistent with the analysis of eosinophil infiltration scores, the expression of most immune checkpoint-related genes is generally higher in the high-risk group than in the low-risk group, such as CD276, CD80 and VTCN1 (Supplementary Figure 5A). We further investigated whether ERS could serve as a predictive indicator for immunotherapy in HCC patients. A negative correlation between TIDE scores and risk scores was observed (Figures 6C, D). TIDE scores above 0 are often considered unfavorable for response to anti-PD1 or anti-CTLA4 immunotherapy and patients in the high-risk group showed higher TIDE scores compared with those in the low-risk group 37. Therefore, it is likely that patients with HCC in the low-ERS will respond to immunotherapy.

### The expression of eosinophil-associated genes is confirmed in HCC samples

**Figure 7:**
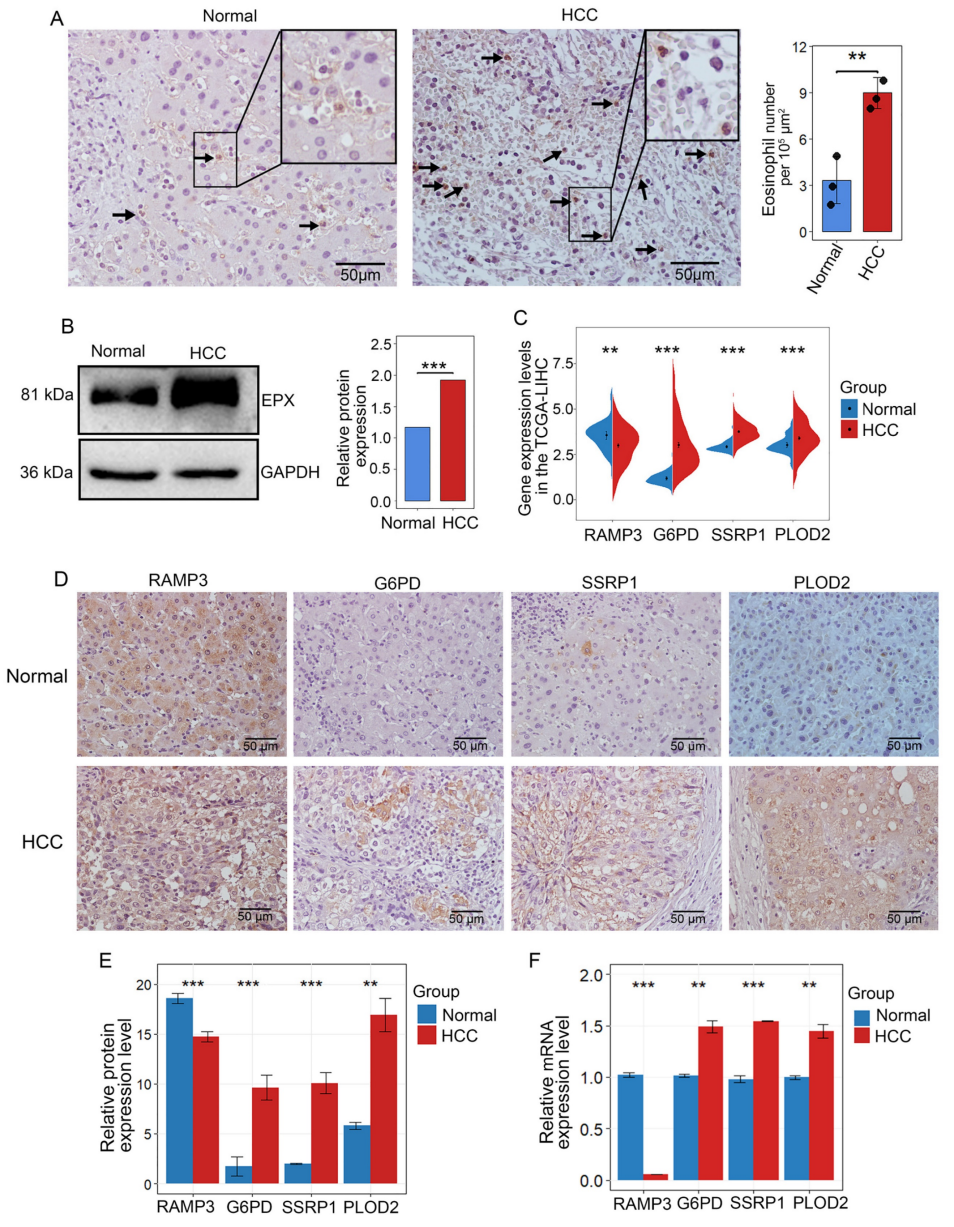
** Multi-level experimental validation of differential gene expression in adjacent and HCC tissues.** (A) Representative images of IHC validation showing the expression of the eosinophil marker gene EPX in adjacent non-tumor tissues and HCC samples. Black arrows indicate positive cells (B) Western blot analysis of the expression of EPX in adjacent and tumor tissues. (C) Differential expression of RAMP3, G6PD, SSRP1 and PLOD2 in adjacent and tumor tissues from the TCGA-LICN database. (D, E) Representative IHC images indicating the expression of RAMP3, G6PD, SSRP1 and PLOD2 in adjacent non-tumor tissues and tumor tissues. Scale bar represents 50 μm. (F) RT-qPCR showing the expression differences of RAMP3, G6PD, SSRP1 and PLOD2. *, P < 0.05; **, P < 0.01; and ***, P < 0.001.

Eosinophil peroxidase (EPX) was used to evaluate the infiltration of eosinophils in HCC 38. IHC results from samples of three stage II HCC patients revealed that eosinophils were primarily located within the HCC and the surrounding connective tissues (Figure 7A and Supplementary Figure 5B). Western blot analysis demonstrated that EPX expression was higher in HCC tissues compared to adjacent non-tumor tissues (Figure 7B). Analysis of the TCGA-LIHC data revealed that in high-risk HCC, RAMP3 expression was downregulated, while the expression of G6PD, PLOD2 and SSRP1 were upregulated (Figure 7C). To confirm these findings, immunohistochemistry showed that compared to adjacent non-tumor tissues, RAMP3 expression was downregulated, whereas the expression of other three genes expression were upregulated in HCC samples (Figures 7D, E). RT-qPCR results corroborated the findings in IHC analysis (Figure 7F). The differential expression of these four genes between HCC and adjacent normal tissues highlights their significance as prognostic markers for HCC.

### IL-33-driven eosinophil recruitment could inhibit intrahepatic tumor development

**Figure 8:**
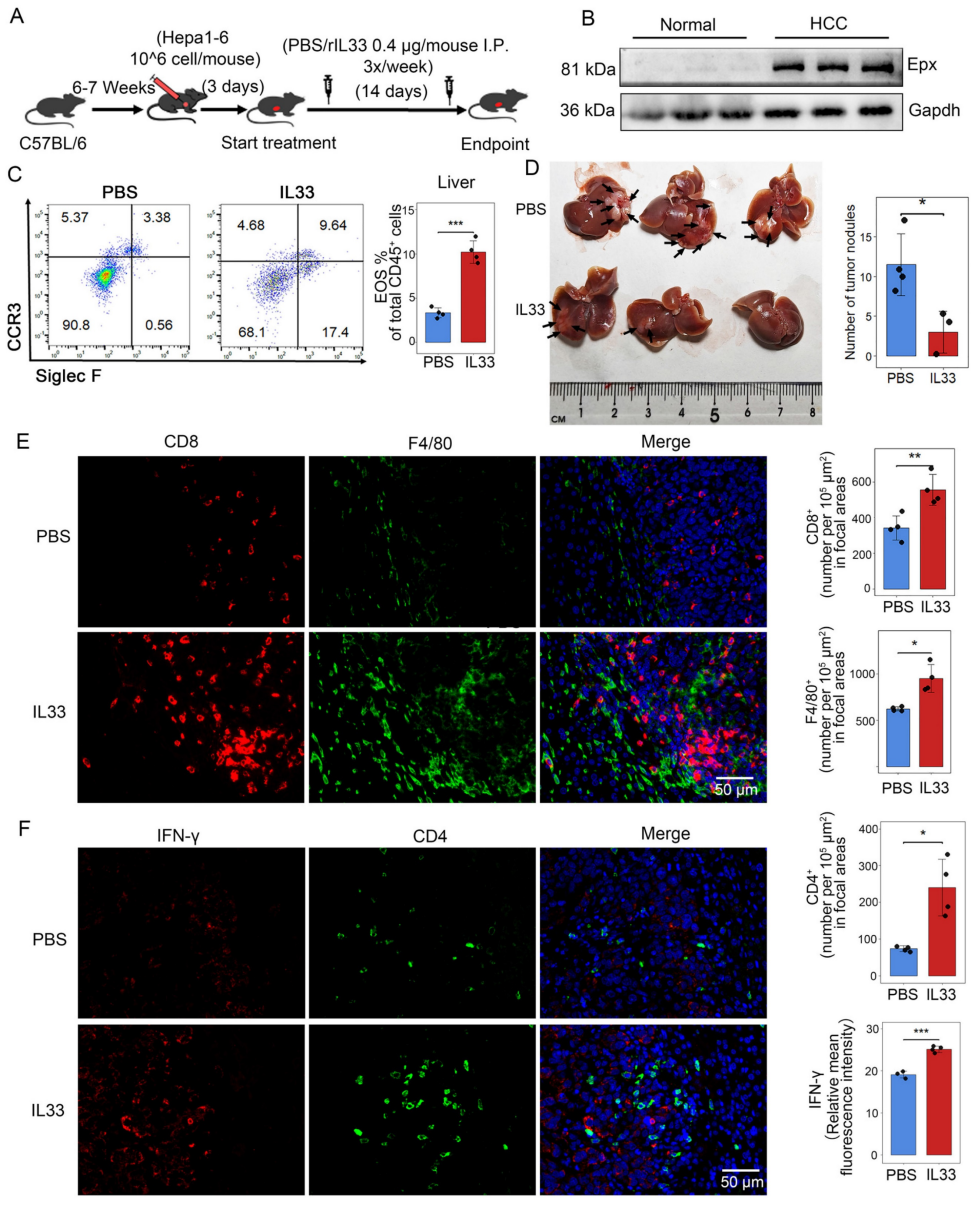
** IL-33 drives recruitment of eosinophils and activates immune cells in HCC.** (A) Male C57BL/6 mice were subjected to intrahepatic injection of Hepa1-6 cells. Three days post-injection, the mice received intraperitoneal injections of IL-33 or PBS three times a week, with a dosage of 0.4 μg per mouse. After 17 days, the mouse livers were collected, the number of liver tumor nodules was counted, and the tissues were embedded (n=7). (B) Eosinophil infiltration in normal mouse liver and HCC tissues was detected via Western blot analysis. (C) Following intraperitoneal injection of rIL-33/PBS, eosinophil infiltration was analyzed using flow cytometry after non-parenchymal cells were extracted from mouse livers. (D) Representative images of mouse liver nodules following rIL-33/PBS treatment. (E) Representative images of immunofluorescence staining of CD8 (red) and F4/80 (green) on mouse liver sections. (F) Representative images of immunofluorescence staining of CD4 (green) and IFN-γ (red) on mouse liver sections. Scale bar represents 50 μm. *, P < 0.05; **, P < 0.01; and ***, P < 0.001.

Just as in human liver tumors, mouse liver also recruits a large number of eosinophils after the injection of Hepa1-6 cells, forming mouse orthotopic liver tumors (Figures 8A, B). Consistently, we found that IL-33 can drive eosinophil infiltration into the liver as reported 39. We hypothesized that intentionally inducing eosinophil accumulation in tumors through rIL-33 might enhance the therapeutic benefits of autoimmunity in cancer. Flow cytometry analysis showed that rIL-33 led to an increase in eosinophils abundance of the liver (Figure 8C). And the number of intrahepatic tumor nodules in mice injected with rIL-33 was significantly reduced (Figure 8D). Moreover, rIL-33-mediated eosinophils promoted the infiltration of CD8^+^ T cells, CD4^+^ T cells and macrophages into tumors (Figures 8E, F), accompanied by a significant increase in expression levels of effector factor IFN-γ. These results revealed that rIL-33 can participate in the recruitment of eosinophils in the liver and enhance the antitumor activity of certain immune cells, indicating the feasibility of using rIL-33-mediated eosinophil recruitment to treat HCC.

## Discussion

HCC is the third most leading cause of cancer death worldwide 40. Currently, the use of systemic therapies, including tyrosine kinase inhibitors (TKIs), immune checkpoint inhibitors (ICIs) and monoclonal antibodies, has significantly improved overall survival and life quality for HCC patients 41. However, monotherapy with ICI provided significant clinical benefit in only about 15-20% of HCC patients who received immune checkpoint therapy 42, 43. The challenge we face is to identify appropriate biomarkers to recognize this subgroup. In melanoma, eosinophils are considered one of the most promising cellular biomarkers for post-ICI cancer treatment and may even be effector cells in the terminal stage, especially for anti-CTLA4 and anti-PD-1 antibodies 44, 45. However, there are currently few studies on the relationship between eosinophil infiltration and prognosis of HCC. We performed ssGSEA analysis on HCC patients from the TCGA database to calculate the infiltration score of eosinophils in HCC, high eosinophil infiltration in HCC patients is associated with longer survival. Some studies in a variety of solid tumors suggest that group 2 Innate Lymphoid Cells (ILC2) in the TME can directly influence the proliferation of eosinophils by releasing type 2 cytokines (IL-4, IL-13 and IL-5) and indirectly mediate the recruitment of eosinophils into the TME 46. Here, we found that eosinophil infiltration may be beneficial for prolonging the survival of HCC patients These results suggest that eosinophils may play an important role in the development, progression and immunotherapy of HCC.

Based on the eosinophil infiltration score grouping, we conducted WGCNA analysis on TCGA-LIHC data to explore the potential role of eosinophil-related genes in HCC patients. After a series of screening, we identified four eosinophil-related genes (RAMP3, G6PD, SSRP1 and PLOD2). RAMP3 protein is known to transport the calcitonin receptor-like receptor (CRLR) to the cell membrane and help CRLR to act as a receptor for adrenomedullin (ADM), calcitonin gene-related peptides (CGRP) and amylin 47, 48. RAMP3 therefore serves as a crucial transducer for several autocrine signals triggered by these peptides. In liver cancer, increased expression of RAMP3 can mitigate the negative effects of TP53 mutations on patient survival 49. In our study, high expression of RAMP3 positively correlated with a good prognosis in HCC. Tumor cells have a high demand for NADPH, which is produced via the PPP pathway 50. G6PD is considered the only enzyme that regulates the pentose phosphate pathway (PPP). Some studies suggest that targeting this requirement by inhibiting G6PD could be a therapeutic strategy for HCC 51. Our analysis consistently indicated a positive correlation between G6PD and poor prognosis in liver cancer.

Structure-Specific Recognition Protein 1 (SSRP1) is a subunit of the Facilitates Chromatin Transcription (FACT) complex, a histone chaperone that plays a key role in DNA replication, repair and transcription 52-55. SSRP1 is reported to be upregulated in many tumors and is associated with a poorer prognosis 56. Consistently, elevated expression of SSRP1 correlated with poor prognosis in HCC. Lysyl hydroxylase 2 (LH2), encoded by the PLOD2 gene, is a key enzyme that mediates collagen cross-linking 57. Some studies suggest that LH2 may promote tumor invasion and metastasis 58, 59. And PLOD2 is highly expressed in tumor tissues compared to normal tissues, including liver cancer 60. In accordance with these, patients with high PLOD2 expression have significantly shorter overall survival. However, the molecular mechanisms underlying the relationship between gene expression and eosinophil infiltration were not elucidated in this study, which will be investigated in the next research.

A prognostic risk model for HCC was constructed using four genes associated with eosinophil infiltration. Multivariate Cox regression analysis of ERS and clinicopathological features showed that ERS and cancer stage are independent prognostic factors for HCC. The ERS model was validated using ROC and calibration curves with GEO data. Compared to similar studies, our risk model achieved a one-year AUC of 0.819, surpassing the macrophage-based models (one-year AUC: 0.706) 61, the neutrophil-based models (one-year AUC: 0.769) 62, and the hypoxia-based models (one-year AUC: 0.73) 63. This indicates that eosinophil infiltration impacts the progression of HCC, and also demonstrates the high efficacy of our prognostic model, which will be validated in real cohorts.

Research has shown that eosinophil depletion markedly reduces the anti-melanoma effects mediated by IL-33, along with the recruitment and activation levels of CD8^+^ T cells and NK cells 64, which suggests that eosinophils can enhance T cell activity, thereby indirectly inhibiting the growth of primary tumors. In our study, the TCGA-LIHC data analysis showed a strong correlation between eosinophil scores and the scores of other immune tissues. Eosinophil infiltration scores revealed significant associations with the expression of immunotherapy response-related genes. The eosinophil infiltration-based risk score model can distinguish patients who respond to anti-PD1 and anti-CTLA4 immunotherapy from those who do not. And the co-operation between eosinophils and T cells enhances the response to ICI therapy 65. The *In vivo* mouse experiments demonstrated that IL-33 could induce the recruitment of eosinophils, CD4^+^ T cells, CD8^+^ T cells, and macrophages to HCC, significantly reducing the number of tumor nodules. In addition, eosinophils may inhibit liver tumor metastasis by enhancing the activity of NK cells 66. In conclusion, eosinophil infiltration in HCC is beneficial for patients receiving immunotherapy.

In summary, we have established and validated a risk score model based on the eosinophil model to predict the overall survival of HCC patients and differentiate sensitivity to immunotherapy between high/low risk groups. Treatment with IL-33 to promote the recruitment of eosinophils can effectively inhibit the progression of liver cancer in mice. Our findings could contribute to a deeper understanding of eosinophil infiltration in HCC and provide a new risk score model for precision medicine to make treatment decisions at different tumor stages of HCC.

## Supplementary Material

Supplementary figures and tables.

## Abbreviations

HCC: hepatocellular carcinoma
TCGA: The Cancer Genome Atlas
ssGSEA: single-sample Gene Set Enrichment Analysis
WGCNA: Weighted Gene Co-expression Network Analysis
TIDE: Tumor Immune Dysfunction and Exclusion
ERS: eosinophil-associated gene risk score
IHC: immunohistochemical
RT-qPCR: real-time quantitative PCR
EPX: eosinophil peroxidase
NK: natural killer
TME: tumor microenvironment
rIL-33: recombinant IL-33
IP: intraperitoneally
GO: Gene Ontology
KEGG: Kyoto Encyclopedia of Genes and Genomes
AUC: the areas under the receiver operating characteristic curves
