# Supplementary material for: Eosinophil-Associated Genes are Potential Biomarkers for Hepatocellular Carcinoma Prognosis
Source: J Cancer. 2024 Sep 3;15(17):5605–21. doi: 10.7150/jca.95138 (PMC11414626; doi:10.7150/jca.95138)
Supplement: Supplementary file 1 — Supplementary figures and tables. [file jcav15p5605s1.pdf]

Eosinophil-Associated Genes are Potential Biomarkers for Hepatocellular Carcinoma Prognosis supplementary material

Qinghao Wang[1,2*], Zixin Zhang[1,2*], Hao Zhou[1], Yanling Qin[1], Jun He[3], Limin Li[2,4†], Xiaofeng Ding[1, 2,5†]

[1] The National & Local Joint Engineering Laboratory of Animal Peptide Drug Development, College of Life Science, Hunan Normal University, Changsha, 410081, China

[2] Institute of Interdisciplinary Studies, Hunan Normal University, Changsha, 410081, China

[3] Hunan Provincial Key Laboratory of Regional Hereditary Birth Defects Prevention and Control, Changsha Hospital for Maternal & Child Health Care Affiliated to Hunan Normal University, Changsha, 410007, China

[4] College of Engineering and Design, Hunan Normal University, Changsha, 410081, China

[5] Peptide and Small Molecule Drug R&D Platform, Furong Laboratory, Hunan Normal University, Changsha, 410081, China

[†] Correspondence: Limin Li, Ph. D., hncslilimin@hunnu.edu.cn and Xiaofeng Ding, Ph. D., dingxiaofeng@hunnu.edu.cn.

*These authors contributed equally: Qinghao Wang and Zixin Zhang.

Supplementary Table 1 Primer pairs in this study

Name	Sequence (5'- to -3')	Purpose
RAMP-F	TAGCAGCCCACGACATTGG	RT-qPCR
RAMP-R	GGGAAGGCTTTCGCAGACAT	
G6PD-F	GTAGTGGTCGATGCGGTAGA	RT-qPCR
G6PD-R	CGAGGCCGTCACCAAGAAC	
SSRP-F	GTCACCTCATTCTTGCCTGTG	RT-qPCR
SSRP-R	AAACTCACTATCGCCTTGAGC	
PLOD-F	AGGGGTTGGTTGCTCAATAAAAA	RT-qPCR
PLOD-R	CATGGACACAGGATAATGGCTG	
GAPDH-F	CTGGAAGATGGTGATGGGATT	RT-qPCR
GAPDH-R	GATTCCACCCATGGCAAATTC	

Supplementary Table 2 Clinical characteristics of HCC patients from the TCGA database

Variables	Level	Overall (total=300)
Age (median [IQR])	61 [52,69]	
Gender (%)	Female	96 (32.0)
	Male	204 (68.0)
Age 60 (%)	<60	135 (45.0)
	>=60	165 (55.0)
History of Drinking (%)	No/unknown	197 (65.7)
	Yes	103 (34.3)
Virus Hepatitis (%)	No/unknown	174 (58.0)
	Yes	126 (42.0)
Pathologic T (%)	T1	147 (49.0)
	T2	74 (24.7)
	T3	65 (21.7)
	T4	11 (3.7)
	TX	1 (0.3)
	Unknown	2 (0.7)
Pathologic stage (%)	Stage I	140 (46.7)
	Stage II	67 (22.3)
	Stage III	71 (23.7)
	Stage IV	1 (0.3)
	Unknown	21 (7.0)
Person neoplasm cancer status (%)	Tumor free	162 (54.0)
	Unknown	11 (3.7)
	With tumor	127 (42.3)
Radiation therapy (%)	No	300 (100.0)
Vital status (%)	Decreased	102 (34.0)
	Living	198 (66.0)

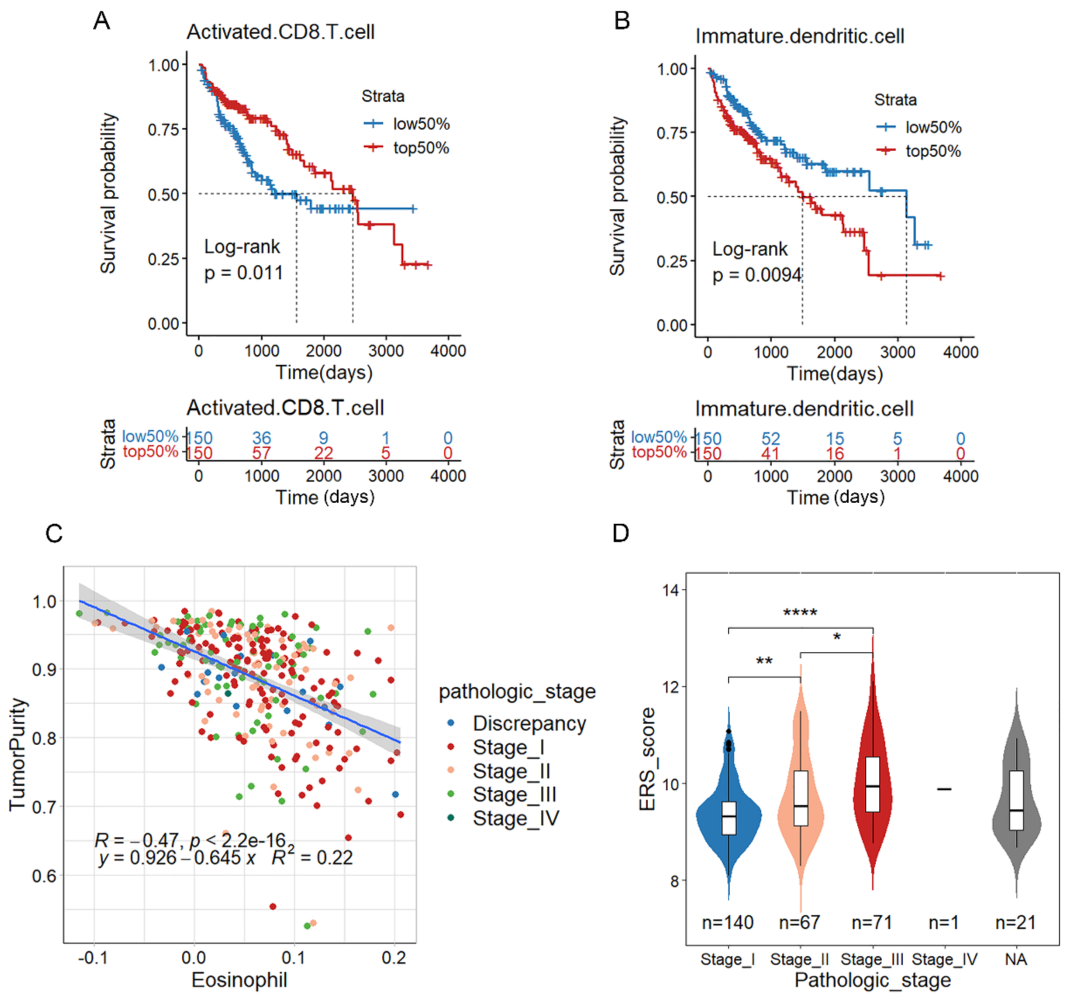

Supplementary Figure 1 The relationship between immune cell infiltration and HCC. (A) Kaplan-Meier survival curves for infiltration with activated CD8 T cells. (B) Kaplan-Meier survival curves for infiltration with immature dendritic cells. (C) Correlation between eosinophil infiltration and tumor purity. (D) ERS risk scores in patients with different pathologic grades.

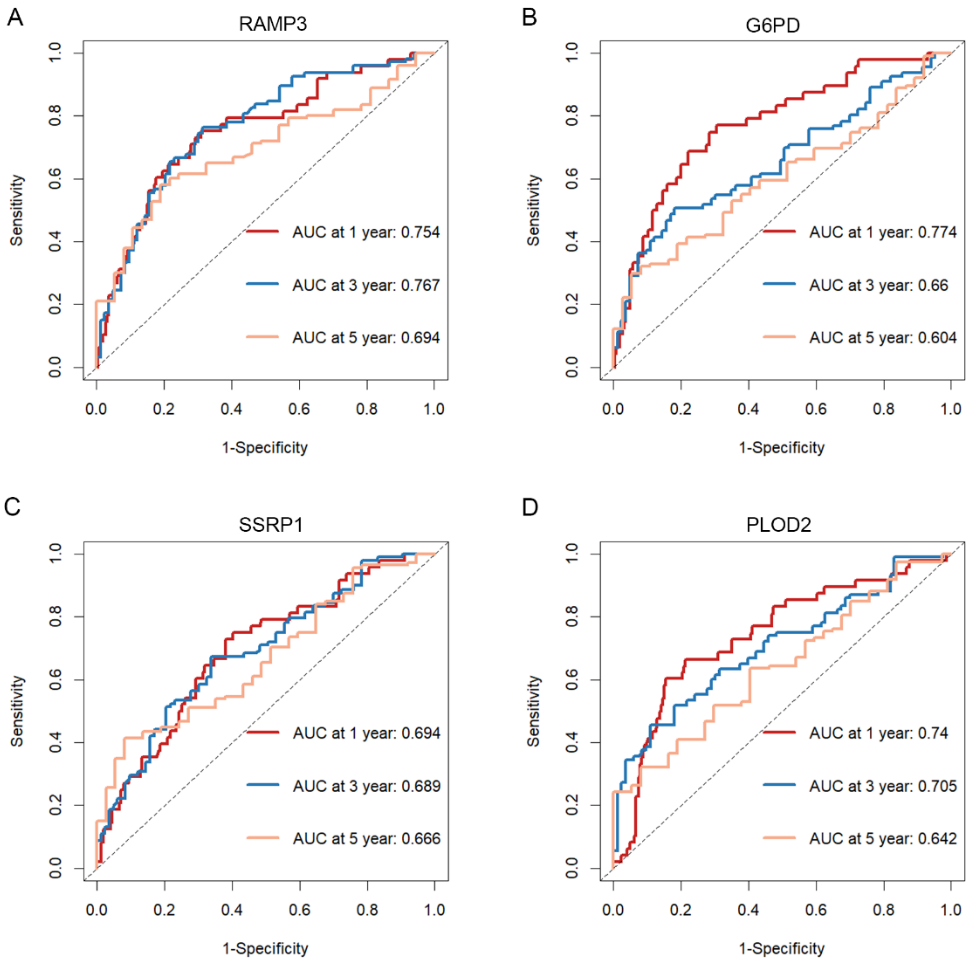

Supplementary Figure 2 Plot the ROC curve of survival rate based on the expression level of a single gene.

(A) ROC curves for 1-, 3-, and 5-year survival according to the expression level of RAMP3. (B) ROC curves for 1-, 3-, and 5-year survival according to the expression level of G6PD. (C) ROC curves for 1-, 3-, and 5-year survival according to the expression level of SSRP1. (D) ROC curves for 1-, 3-, and 5-year survival according to the expression level of PLOD2. AUC, area under the curve.

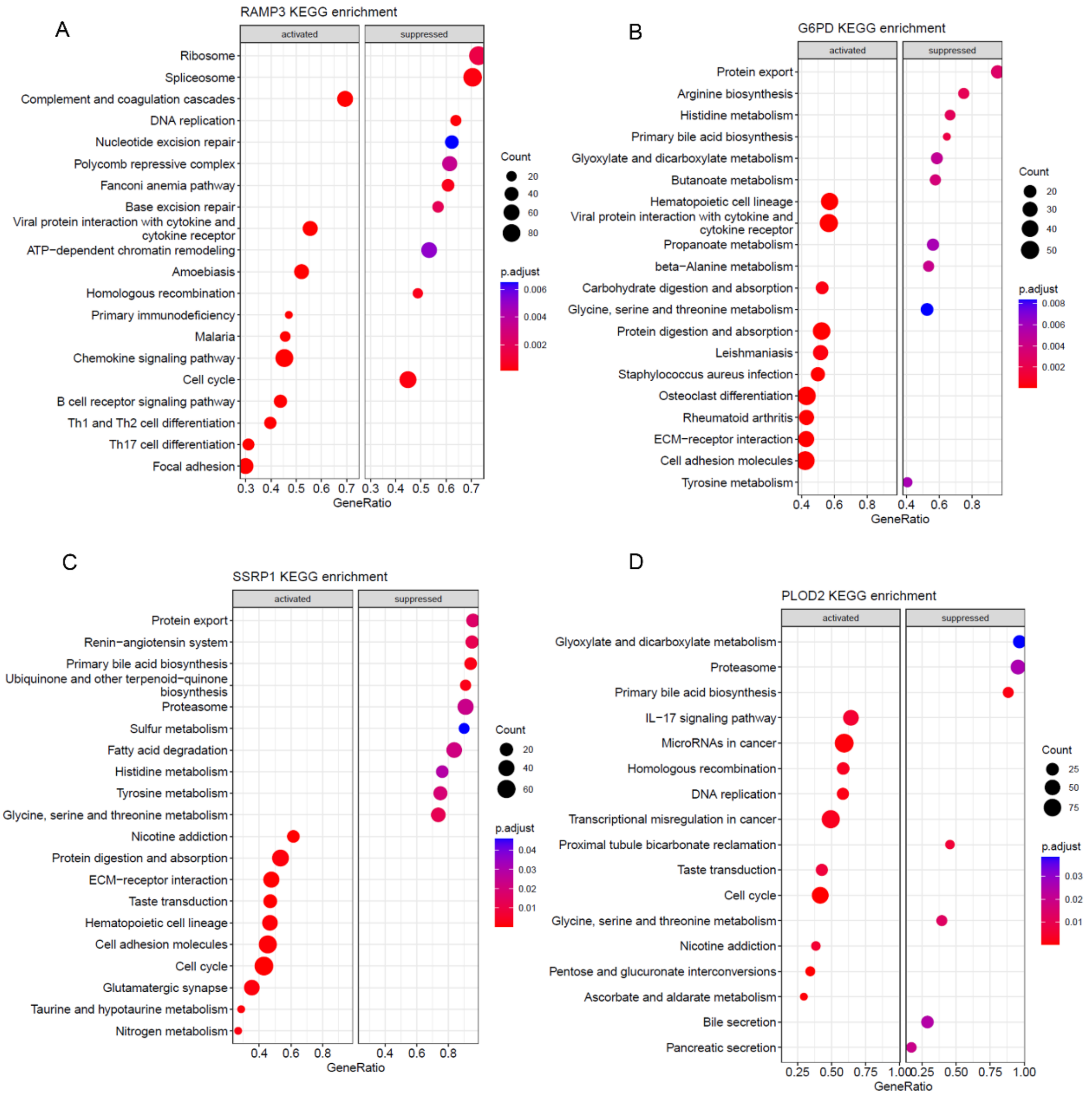

Supplementary Figure 3 Grouping based on the expression levels of relevant genes and conducting KEGG analysis using bubble plots.

(A-D) Based on the expression of the correlated genes, patients were divided into two groups, a high expression group and a low expression group, with bubble charts for KEGG analysis.

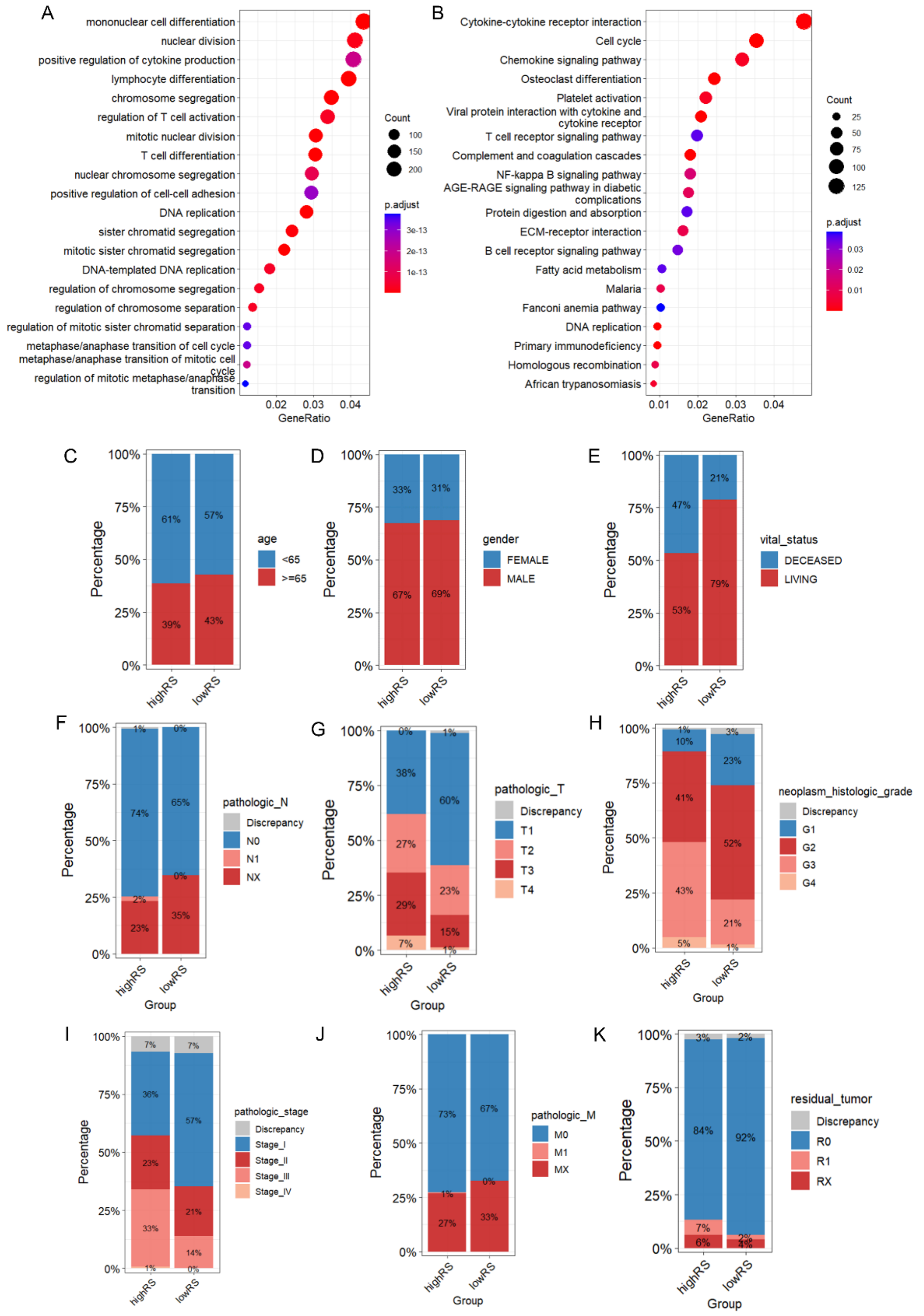

Supplementary Figure 4 GO analysis, KEGG analysis, and distribution of clinical case characteristics of patients.

(A-B) Patients were divided into high-risk and low-risk groups based on eosinophil infiltration scores. Bubble chart for GO analysis and bubble chart for KEGG analysis. (C-K) Distribution and percentage of clinical case characteristics in the ERS high group and ERS low group patients.

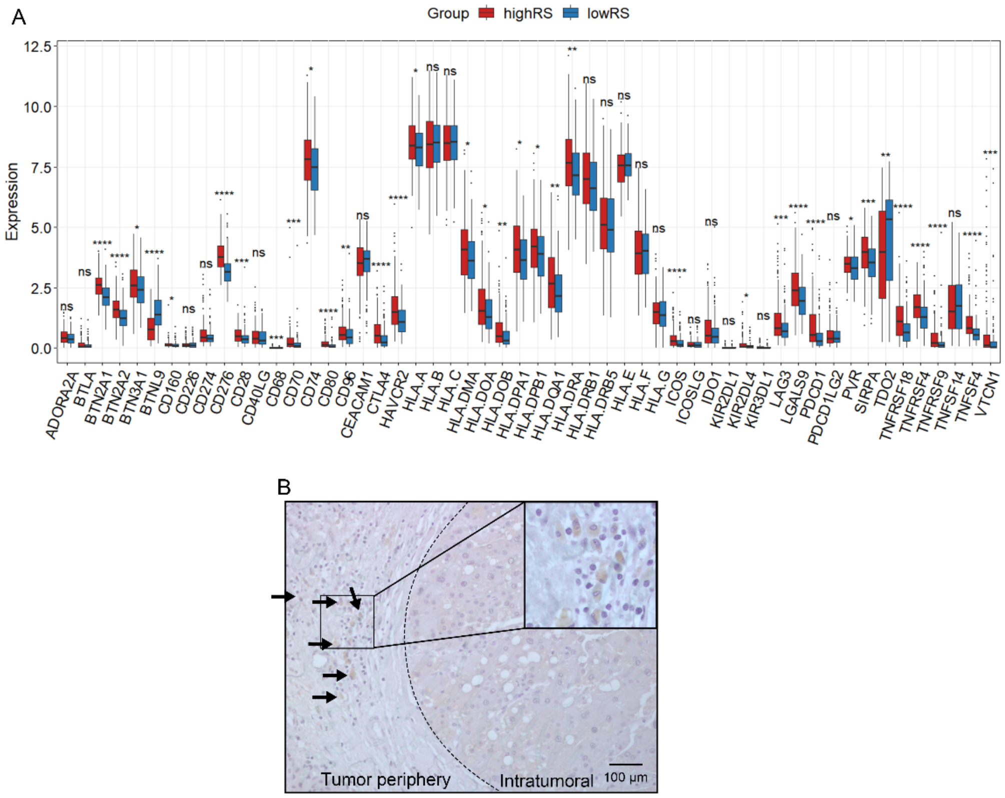

Supplementary Figure 5 The relationship between ERS model score and immune therapy response, and the distribution of EPX in HCC samples.

(A) The relationship between ERS model scores and the expression of genes associated with response to immunotherapy. (B) Representative IHC images showing the distribution of the eosinophil marker gene EPX in adjacent non-tumor tissues and HCC samples. Black arrows indicate positive cells.
